# Modulation of macrophage antitumor potential by apoptotic lymphoma cells

**DOI:** 10.1038/cdd.2016.132

**Published:** 2017-02-03

**Authors:** Jorine J L P Voss, Catriona A Ford, Sofia Petrova, Lynsey Melville, Margaret Paterson, John D Pound, Pam Holland, Bruno Giotti, Tom C Freeman, Christopher D Gregory

**Affiliations:** 1 Medical Research Council (MRC) Center for Inflammation Research, Queen's Medical Research Institute, University of Edinburgh, Edinburgh EH16 4TJ, UK; 2 The Roslin Institute, R(D)SVS, University of Edinburgh, Easter Bush EH25 9RG, UK

## Abstract

In aggressive non-Hodgkin's lymphoma (NHL), constitutive apoptosis of a proportion of the tumor cell population can promote net tumor growth. This is associated with the accumulation of tumor-associated macrophages (TAMs) that clear apoptotic cells and exhibit pro-oncogenic transcriptional activation profiles characteristic of reparatory, anti-inflammatory and angiogenic programs. Here we consider further the activation status of these TAMs. We compare their transcriptomic profile with that of a range of other macrophage types from various tissues noting especially their expression of classically activated (IFN-*γ* and LPS) gene clusters – typically antitumor – in addition to their previously described protumor phenotype. To understand the impact of apoptotic cells on the macrophage activation state, we cocultured apoptotic lymphoma cells with classically activated macrophages (M_(IFN-*γ*/LPS)_, also known as M1, macrophages). Although untreated and M_(IFN-*γ*/LPS)_ macrophages were able to bind apoptotic lymphoma cells equally well, M_(IFN-*γ*/LPS)_ macrophages displayed enhanced ability to phagocytose them. We found that direct exposure of M_(IFN-*γ*/LPS)_ macrophages to apoptotic lymphoma cells caused switching towards a protumor activation state (often referred to as M2-like) with concomitant inhibition of antitumor activity that was a characteristic feature of M_(IFN-*γ*/LPS)_ macrophages. Indeed, M_(IFN-*γ*/LPS)_ macrophages exposed to apoptotic lymphoma cells displayed increased lymphoma growth-promoting activities. Antilymphoma activity by M_(IFN-*γ*/LPS)_ macrophages was mediated, in part, by galectin-3, a pleiotropic glycoprotein involved in apoptotic cell clearance that is strongly expressed by lymphoma TAMs but not lymphoma cells. Intriguingly, aggressive lymphoma growth was markedly impaired in mice deficient in galectin-3, suggesting either that host galectin-3-mediated antilymphoma activity is required to sustain net tumor growth or that additional functions of galectin-3 drive key oncogenic mechanisms in NHL. These findings have important implications for anticancer therapeutic approaches aimed at polarizing macrophages towards an antitumor state and identify galectin-3 as a potentially important novel target in aggressive NHL.

Tumor-associated macrophages (TAMs) form an integral part of the microenvironment of many cancers. Despite their innate antitumor potential, TAMs have been associated with poor prognosis in most cases and have been shown to adopt anti-inflammatory, prorepair phenotypes, promoting angiogenesis, matrix remodeling, tumor growth and metastasis.^1, 2^ TAMs, however, can engage in antitumor activities, effectively constraining growth of nascent and of certain established tumors.^3, 4^ Furthermore, human cancer gene array analyses reveal macrophage signatures containing, in addition to tumor-promoting gene activation, potential antitumor pathways such as MHC class II antigen presentation and T-cell costimulation.^5^ This reflects the plasticity of TAMs in response to the tumor microenvironment.

TAMs display highly active phagocytic and lipid metabolic pathways suggestive of their homeostatic activity in the clearance of dying cells that are constitutively produced during net tumor growth.^5, 6^ Cell death is often a prominent feature of malignant disease, tumor growth resulting from the imbalance between proliferation and death.^7^ Indeed, apoptosis and caspase activation have been found to correlate with aggressive disease in multiple malignancies,^8, 9, 10, 11, 12, 13^ including ‘starry-sky' lymphoma^6^ (classically Burkitt's lymphoma, BL) in which TAMs engaged in the clearance of apoptotic cells appear analogous to stars in a sky of tumor cells.^14, 15^ Timely and effective clearance of apoptotic cells by macrophages prevents the release of potentially toxic or immunogenic intracellular contents from dying tumor cells, and may contribute to suppression of inflammation and inhibition of innate and adaptive antitumor immunity. Engagement with apoptotic tumor cells may also promote angiogenic and matrix remodeling signaling in TAMs, extending the known protumor capacities of the apoptotic cell clearance response.^6^

As apoptotic cells can promote oncogenic signaling in macrophages, we hypothesized that interaction and engulfment of apoptotic tumor cells could help counter potential antitumor signaling properties of the tumor microenvironment, biasing the TAM phenotype towards tumor support. Here we show that starry-sky TAMs (SS-TAMs) from BL xenografts, previously reported to display an overtly pro-oncogenic transcriptional activation profile (‘M2-like'),^6^ also express genes associated with macrophages activated by the classical stimuli, interferon-*γ* (IFN-*γ*) and lipopolysaccharide (LPS), which typically have antitumor activity (M1 activation state). Such macrophages were found to interact effectively with apoptotic tumor cells, resulting in switching of activation state from antitumor to protumor with concomitant suppression of tumor cell killing activity and promotion of net tumor population growth.

## Results

### Characteristics of classically activated macrophages can be identified in SS-TAMs and other macrophage subtypes

Given that the core macrophage signature of six large human cancer data sets contained classically activated characteristics,^5^ we further analyzed *in situ* transcriptomics data sets of laser-microdissected SS-TAMs from BL xenografts (as described previously^6^), seeking signatures of classical activation. These data provide detailed, unbiased *in situ* activation profiles of SS-TAMs, which are actively engaged in the clearance of apoptotic BL cells. Functional annotation using the Database for Annotation and Integrated Discovery (DAVID) of all genes upregulated in SS-TAMs, compared with germinal center macrophages (GCMs) revealed that in addition to the previously reported tumor-promoting pathways,^6^ genes associated with immune and inflammatory responses linked to classical activation were also upregulated (Table 1).

**Figure 1:**
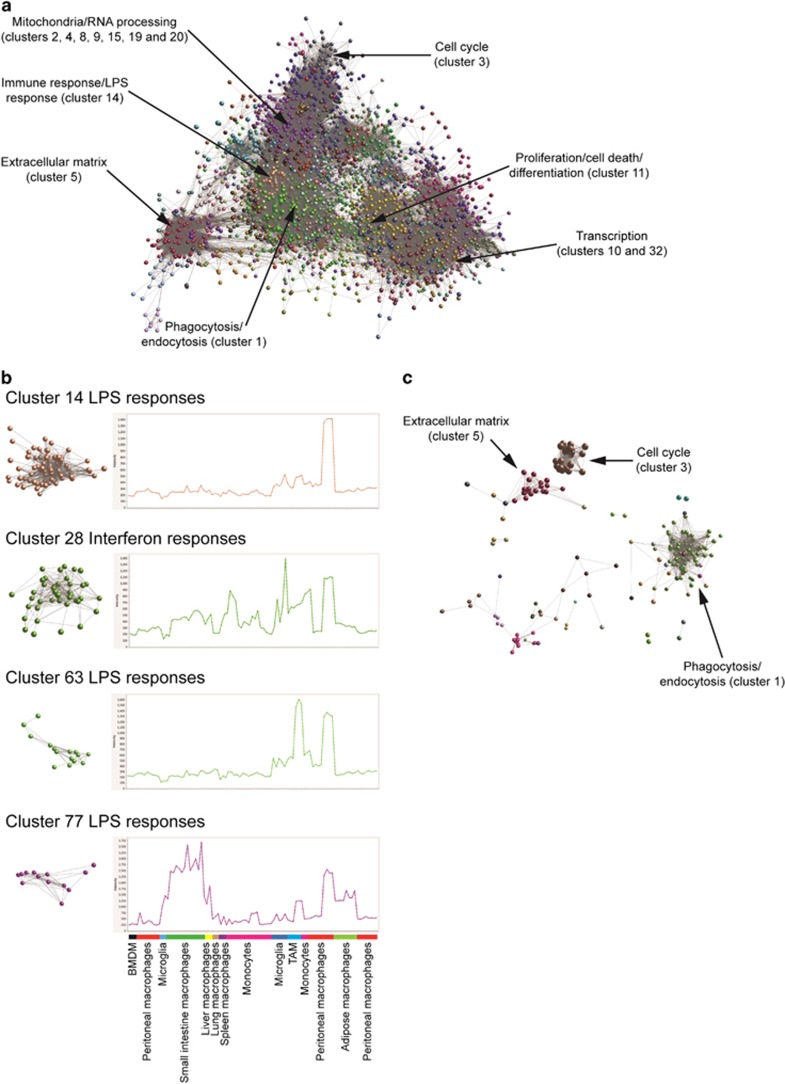
Characteristics of classically activated macrophages can be identified in SS-TAMs and other macrophage subtypes. Ninety samples, representing 29 mouse macrophage and monocyte populations from 6 individual studies were normalized and the tool BioLayout Express3D was used to calculate pairwise Pearson's correlation coefficients for every transcript represented on the array. A network graph was constructed using a cutoff of *r*=0.8, where nodes represent transcripts analyzed on the array, and edges represent the coexpression relationships between the transcripts. The resultant graph was laid out in three-dimensional space and comprised of 8022 nodes (transcripts), connected by 215 224 edges. The graph was then clustered using the MCL algorithm with an inflation value of 2.2. This resulted in 1061 clusters containing more than five nodes. (**a**) The individual nodes (transcripts) in each cluster color-coded, and the functional association of the genes in some of the larger clusters is indicated. (**b**) Examples of clusters associated with the activation of macrophages by LPS are shown isolated from the main graph alongside their average expression profile of the transcripts that make up the cluster. (**c**) Transcripts that are upregulated by SS-TAM (compared with GCM and LNM) were imported and overlaid onto the macrophage data set network graph shown in (**a**) and the main function of some of the larger clusters is indicated

To understand further the activation characteristics of SS-TAM data sets, we analyzed the transcriptomic profiles of a range of monocyte and macrophage data sets selected from the Gene Expression Omnibus (GEO) data repository. Suitable data sets were used to construct the large networks shown in Figure 1a. Cluster analysis using the Markov Clustering (MCL) algorithm^5^ defined clusters of highly connected nodes (transcripts), with edges representing the relationships between the nodes. The main function of each cluster was assessed using gene functional annotation analysis and the main function of the transcripts in some of the largest clusters is indicated. Consistent with the main observed function of macrophages, and previous findings,^16^ the largest cluster of genes identified was associated with endocytosis and phagocytosis (Cluster 1). Other large clusters were found to be associated with other broad biological processes, including mitochondria, ribosomes and RNA processing, cell cycle, extracellular matrix, regulation of differentiation, transcription, proliferation and cell death (Figure 1a). Interestingly, as with SS-TAMs, LPS and IFN response signatures were associated with most macrophages in the data sets (Figures 1a and b).

Examination of the genes upregulated in SS-TAMs as compared with GCMs and unstimulated lymph node macrophages^6^ showed that many were related to endocytosis and phagocytosis, the cell cycle and extracellular matrix clusters (Figure 1c). This is consistent with our previous findings that SS-TAMs are highly phagocytic towards apoptotic tumor cells, have been shown to proliferate *in vivo* and upregulate various matrix metalloproteinases.^6^

### Classically activated macrophages have enhanced phagocytic capacity

**Figure 2:**
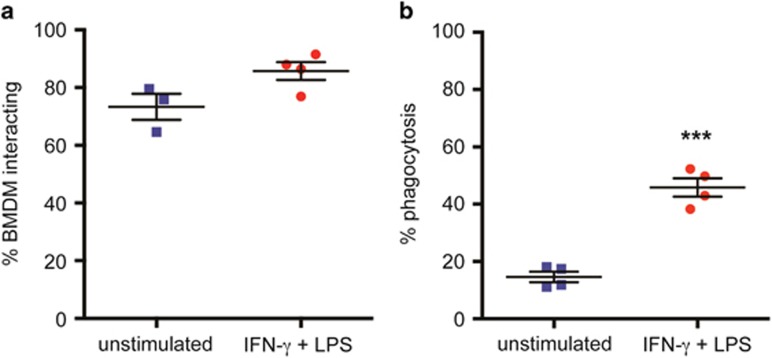
Classically activated (M_(IFN-*γ*/LPS)_) macrophages have enhanced phagocytic capacity. Untreated and IFN-*γ*+LPS-stimulated BMDMs were assessed for their ability to (**a**) interact with and (**b**) phagocytose apoptotic BL2 cells in a microscopy-based phagocytosis assay. Data are means±S.E.M. for the percentage of BMDMs demonstrating interaction (*n*=3) or phagocytosis (*n*=4) of apoptotic BL2 cells and untreated controls. Statistical analysis was performed using a two-tailed paired *t*-test. ***P<0.001

To understand their impact on the macrophage activation state, we studied the interaction of apoptotic lymphoma cells with classically activated (IFN-*γ* and LPS) bone marrow-derived macrophages (BMDMs) *in vitro*. Following recent guidelines,^17^ we refer to these macrophages here as M_(IFN-*γ*/LPS)_ macrophages. First, the ability of M_(IFN-*γ*/LPS)_ macrophages to bind to and to phagocytose apoptotic lymphoma cells was investigated. No significant differences were found in the ability of M_(IFN-*γ*/LPS)_ macrophages to bind to apoptotic lymphoma cells, but a significantly larger proportion of M_(IFN-*γ*/LPS)_ macrophages were capable of phagocytosing apoptotic lymphoma cells compared with untreated BMDMs (Figures 2a and b).

### Exposure of M_(IFN-*γ*/LPS)_ macrophages to apoptotic lymphoma cells causes activation switching towards SS-TAMs

**Figure 3:**
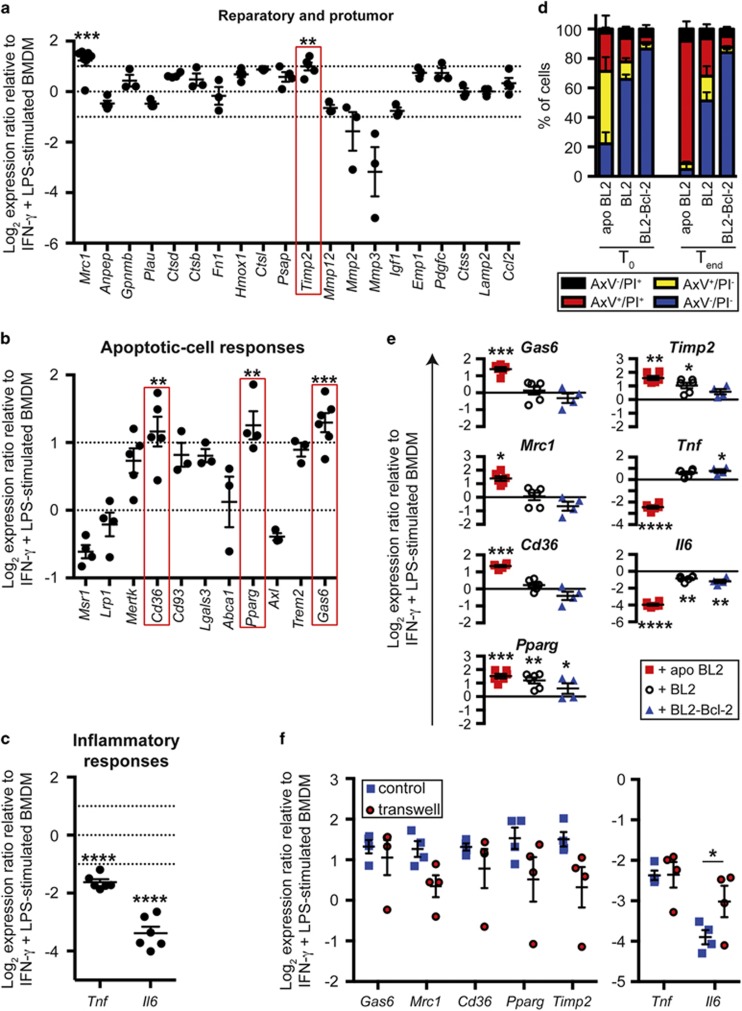
Exposure of M_(IFN-*γ*/LPS)_ macrophages to apoptotic lymphoma cells causes activation switching towards SS-TAMs. Four-hour IFN-*γ*+LPS-stimulated BMDMs were cocultured for 24 h with apoptotic (apo BL2), untreated BL2 (BL2) or untreated *Bcl-2*-transfected BL2 (BL2-Bcl-2) cells. Apoptosis of the BL2 cells was triggered by 100 mJ/cm^2^ UVB irradiation. (**a–f**) mRNA expression of selected genes was assessed following coculture and normalized using the reference genes *Tuba1b*, *Hsp90*, *Hprt* and *B2m*. Expression is presented as log_2_ ratio relative to IFN-*γ*+LPS-stimulated BMDMs. (**a**–**c**) Means±S.E.M. for three to seven independent experiments. The dotted lines at y=1 and y=−1 indicate a twofold increase or decrease of expression, respectively. Statistical analysis was performed on raw data for means showing a twofold increase or decrease of expression, using a two-tailed paired *t*-test and compared IFN*γ*+LPS-stimulated C57BL/6 BMDM cultured with or without apoptotic BL2 cells. **P<0.01, ***P<0.001 and ****P<0.0001. (**d**) Viability of coculture lymphoma cells was assessed by AxV/PI staining at the beginning of coculture (*T*_0_) and at the end of coculture (*T*_end_). (**e**) Data are means±S.E.M. for *n*=6 for all treatments, except *n*=4 for BL2-Bcl-2 cells. Statistical analysis was performed on the raw data using one-way analysis of variance (ANOVA) with Dunnett's multiple comparisons test and is shown compared with IFN-*γ*- and LPS-stimulated Balb/c BMDM. *P<0.05, **P<0.01, ***P<0.001 and ****P<0.0001. (**f**) Balb/c BMDM and lymphoma cells were separated by a 0.44 *μ*m membrane during coculture. Data are means±S.E.M. for *n*=4 for all treatments and expression of IFN-*γ*+LPS-stimulated BMDMs cocultured with apoptotic lymphoma cells is presented as log_2_ ratio relative to IFN-*γ*+LPS-stimulated BMDMs alone. Statistical analysis was performed on the raw data using a two-tailed paired *t*-test. *P<0.05

We next investigated whether apoptotic lymphoma cells could alter the activation state of M_(IFN-*γ*/LPS)_ macrophages from anti- to protumor. To this end, M_(IFN-*γ*/LPS)_ macrophages were cocultured with apoptotic lymphoma cells, and the expression of protumor signatures associated with SS-TAMs as described previously,^6^ together with expression of *Il6* and *Tnf*, markers of M_(IFN-*γ*/LPS)_ macrophage activation, were analyzed. Coculture of M_(IFN-*γ*/LPS)_ macrophages with apoptotic lymphoma cells significantly increased the expression of *Mrc1, Timp2, Cd36, Pparg* and *Gas6*, whereas the expression of *Tnf* and *Il6* were significantly decreased (Figures 3a–c). Next, we investigated whether these differences were due specifically to coculture with apoptotic cells or if coculture with untreated viable lymphoma cells could produce a similar effect. Additionally, as the lymphoma cells show relatively high levels of spontaneous apoptosis (Figure 3d), cocultures were performed with *Bcl-2-*transfected lymphoma cells, whose ability to undergo apoptosis is suppressed.^18^ The viability of the apoptotic and viable lymphoma cells at the start and end of the coculture period is also shown in Figure 3d. As shown in Figure 3e, the upregulation of *Gas6, Mrc1, Cd36* and *Timp2* expression by M_(IFN-*γ*/LPS)_ macrophages was specific for coculture with apoptotic cells, as coculture with unstimulated or *Bcl-2-*transfected lymphoma cells did not lead to a significant upregulation of these genes. Only in the case of *Timp2* could untreated lymphoma cell cultures, of which approximately half the cells would undergo apoptosis during the course of the assay (Figure 3d), also upregulate *Timp2* expression. By contrast, Bcl-2-transfected cells could not (Figure 3e). *Pparg* was significantly upregulated by coculture with both apoptotic and viable lymphoma cells, but there appeared to be a trend of higher upregulation when lymphoma coculture cells displayed higher levels of apoptosis. Additionally, downregulation of *Tnf* was specific for apoptotic, but not viable, lymphoma cells (Figure 3e). Moreover, untreated and *Bcl-2*-transfected lymphoma cells were even capable of significantly upregulating the expression of *Tnf* by M_(IFN-*γ*/LPS)_ macrophages. Coculture with untreated BL2 cells or *Bcl-2*-transfected cells could also significantly decrease expression of *Il6*, but a trend towards a greater inhibition with greater numbers of apoptotic cells was observed (Figure 3e).

### Apoptotic cell-mediated suppression of M_(IFN-*γ*/LPS)_ macrophage gene activation is dependent on factors released from apoptotic cells

Next, we investigated whether the effects of apoptotic cells on gene expression by M_(IFN-*γ*/LPS)_ macrophages was dependent on direct contact. To this end, M_(IFN-*γ*/LPS)_ macrophages were cocultured either directly with apoptotic or untreated lymphoma cells in the same well, or the macrophages and lymphoma cells were separated by a 0.44 *μ*m pore-size transwell membrane, which prevents direct intercellular interactions. No significant differences in expression were observed for *Gas6, Mrc1, Cd36, Pparg* or *Timp2* between cocultures with or without the membrane, suggesting that the effects of coculture with apoptotic cells were due to release of subcellular material from apoptotic cells (Figure 3f). Furthermore, while expression of *Tnf* was similarly unaffected by the separation of M_(IFN-*γ*/LPS)_ macrophages and apoptotic cells, expression of *Il6* was partially inhibited (Figure 3f), suggesting that maximal apoptotic cell-mediated reduction in *Il6* expression by M_(IFN-*γ*/LPS)_ macrophages results from a combination of both direct contact with apoptotic cells and via subcellular material.

### Exposure of M_(IFN-*γ*/LPS)_ macrophages to apoptotic lymphoma cells stimulates lymphoma growth

**Figure 4:**
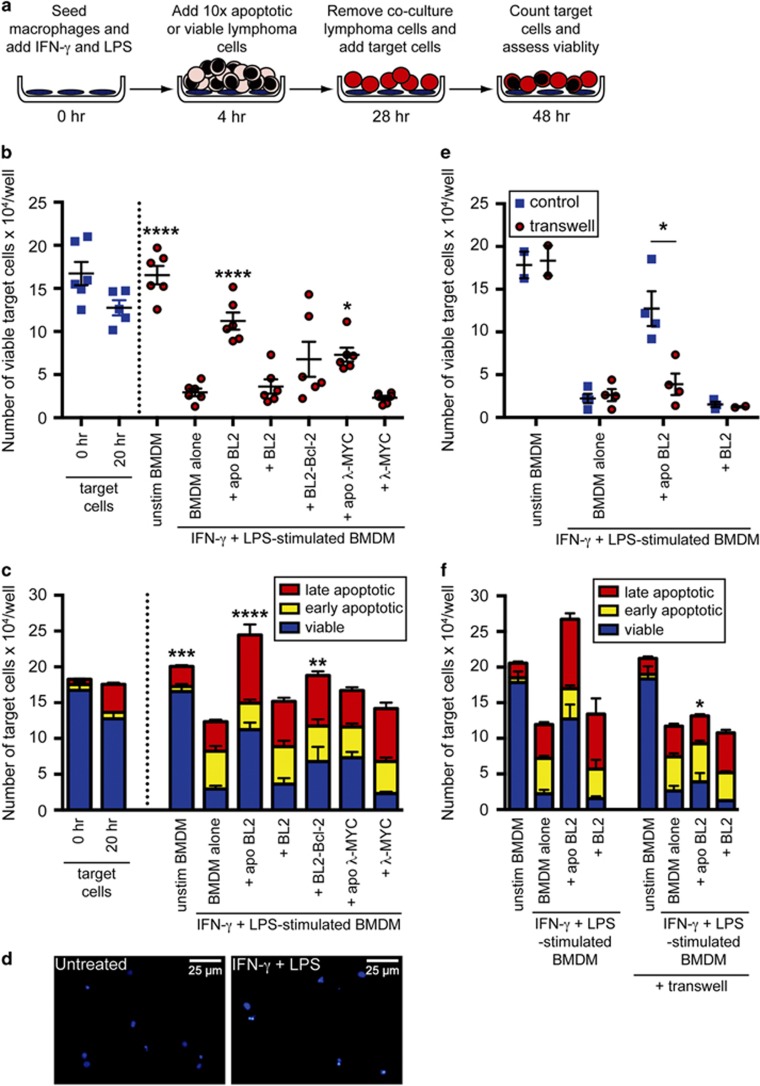
Exposure of M_(IFN-*γ*/LPS)_ macrophages to apoptotic lymphoma cells stimulates lymphoma growth. (**a**) Schematic drawing of macrophage cytotoxicity assay. Balb/c BMDMs were labeled with a PKH67 fluorescent green marker and then activated with IFN-*γ*+LPS or left untreated for 4 h, before apoptotic or viable BL2 or BL2-Bcl-2 human coculture lymphoma cells or apoptotic or viable *λ*-MYC murine coculture lymphoma cells were added for an additional 24 h. After 28 h total pre-treatment, cocultured cells or media controls were removed by repeated washing and macrophage cytotoxicity was tested by the addition of BL2 lymphoma target cells that had been labeled with the fluorescent red membrane marker PKH26 at a 1:1 ratio to BMDMs. Cells were cocultured for 20 h, and at the end of the assay, the viability and number of lymphoma target cells was assessed by flow cytometry using a ratiometric asymmetry probe in combination with a dead cell stain. (**b**) Viable or (**c**) total number of target cells are shown for target cells cultured alone or cultured in the presence of unstimulated or IFN-*γ*+LPS-stimulated BMDMs with or without additional pre-treatments with apoptotic or viable lymphoma cells. Data are mean±S.E.M. for *n*=6. Statistical analysis was performed using one-way analysis of variance (ANOVA) with Dunnett's multiple comparison test and all coculture samples were compared with IFN-*γ*+LPS-stimulated BMDMs alone. *P<0.05, **P<0.01, ***P<0.001 and ****P<0.0001. (**d**) DAPI (4′,6-diamidino-2-phenylindole) staining of lymphoma target cells in untreated and IFN-*γ*+LPS-stimulated BMDM-target cell cocultures showing coculture with IFN-*γ*+LPS-stimulated BMDMs led to condensation of the nuclei of most target cells. (**e**) Viable target cell counts and (**f**) total cell counts of untreated BMDMs and IFN-*γ*+LPS-stimulated cells pre-treated with or without apoptotic or viable BL2 cells either directly (control) or separated by a 0.44 *μ*m transwell. Data are means+S.E.M. for *n*=4. Statistical analysis is shown comparing each of the prestimuli between with or without transwell using an unpaired *t*-test and correcting for multiple comparisons using the Holm–Sidak method. *P<0.05

It has long been known that classically activated macrophages display antitumor cytotoxic and cytostatic effects^19, 20, 21^ and it has previously been shown that coculture with apoptotic cells can reduce the ability of classically activated BMDMs to induce cell death.^22^ To determine the effects of apoptotic lymphoma cell-conditioned M_(IFN-*γ*/LPS)_ macrophages on the lymphoma cell-birth/cell-death equation, we designed the protocol summarized in Figure 4a. Briefly, fluorescently labeled M_(IFN-*γ*/LPS)_ macrophages were cocultured with apoptotic or viable lymphoma cells or media alone. After washing, the macrophages were cocultured with fluorescently labeled viable lymphoma target cells, whose viable, early apoptotic and late apoptotic cell numbers were ultimately assessed as end points using flow cytometry. The results indicate that, compared with untreated BMDMs, M_(IFN-*γ*/LPS)_ macrophages caused significant reduction in the number of viable target cells over a 20h coculture period (Figures 4b and c); target lymphoma cells underwent apoptosis (Figure 4c and d). When M_(IFN-*γ*/LPS)_ macrophages were treated with apoptotic lymphoma cells (either human or murine) before coculture with target lymphoma cells, viable target cell numbers were significantly larger compared with untreated M_(IFN-*γ*/LPS)_ macrophages. This was specific for pre-treatment with apoptotic lymphoma cells, as viable or apoptosis-suppressed lymphoma cells did not significantly affect target cell numbers (Figures 4b and c). Interestingly, the total number of apoptotic and necrotic target cells did not decrease during the course of the coculture (Figure 4c). These results suggest that pre-treatment of M_(IFN-*γ*/LPS)_ macrophages with apoptotic cells stimulate total tumor cell growth in the face of cell destruction.

### The ability of apoptotic cells to stimulate macrophages to promote lymphoma cell growth is contact-dependent

Next, we investigated whether the ability of M_(IFN-*γ*/LPS)_ macrophages pre-treated with apoptotic cells to promote tumor cell growth is contact-dependent. M_(IFN-*γ*/LPS)_ macrophages were either pre-treated directly with apoptotic or untreated BL2 cells, or the two cell types were separated by a 0.44 *μ*m transwell membrane, preventing direct contact. The presence of the membrane during pre-treatments of M_(IFN-*γ*/LPS)_ macrophages with apoptotic cells significantly reduced the number of viable target cells present at the end of the assay (Figure 4e). The total number of apoptotic cells was not affected (Figure 4f). This indicates that the tumor cell growth-promoting effects of apoptotic lymphoma cell-treated M_(IFN-*γ*/LPS)_ macrophages are dependent on direct contact between macrophages and apoptotic cells.

### Galectin-3 deficiency attenuates antilymphoma activity by M_(IFN-*γ*/LPS)_ macrophages

**Figure 5:**
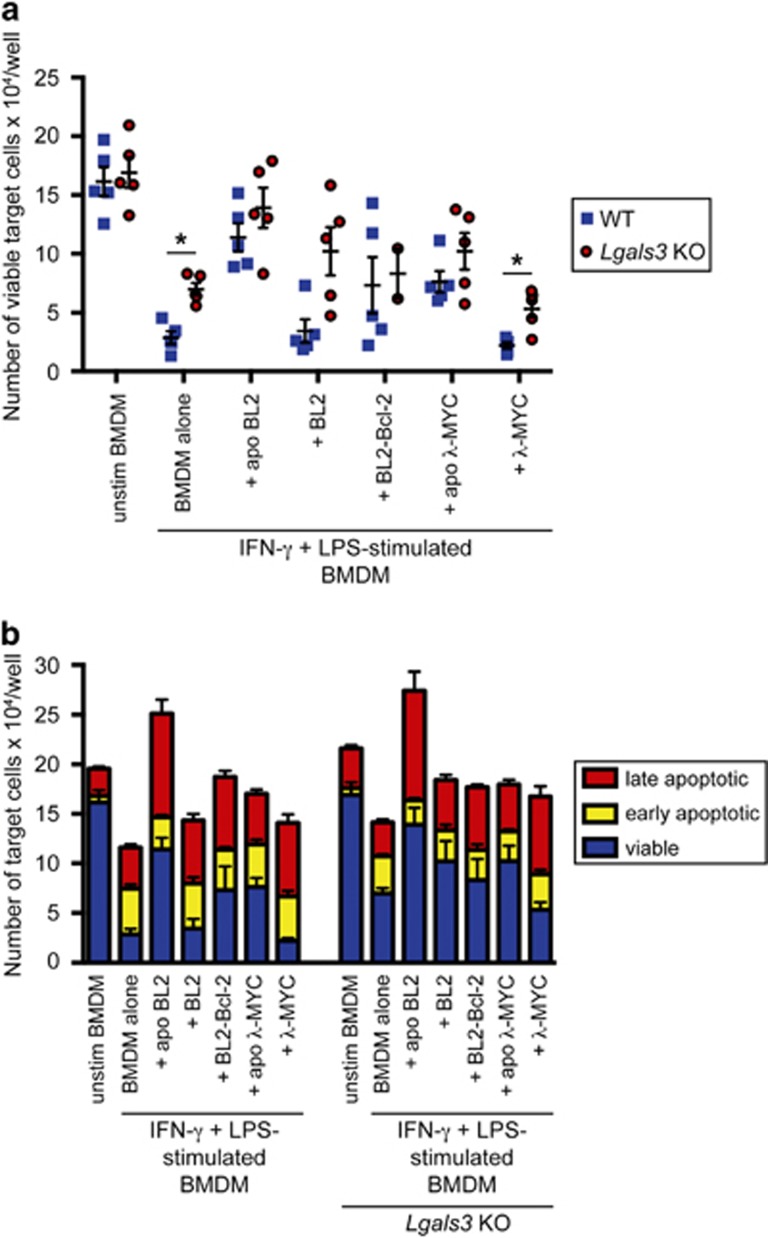
Galectin-3 deficiency attenuates antilymphoma activity by M_(IFN-*γ*/LPS)_ macrophages. Mature BMDMs from WT or *Lgals3* KO mice were pre-treated with IFN-*γ*+LPS for 4 h before coculture with 10x the number of apoptotic or untreated lymphoma cells. The ability of the macrophages to kill target lymphoma cells was then measured by the addition of an equal number of lymphoma cells. Viable target cell counts (**a**) and total cell counts (**b**) of untreated BMDMs and IFN-*γ*+LPS-stimulated cells cocultured with or without apoptotic or viable lymphoma cells are shown. Data are means±S.E.M. for *n*=4. Statistical analysis was performed on the raw data using an unpaired *t*-test and corrected for multiple comparisons using the Holm–Sidak method. *P<0.05

We next attempted to define further the mechanism underlying the protumor effects of apoptotic lymphoma cells interacting with antitumor macrophages. We reasoned that the protein lectin, galectin-3 may be active in this context as (1) it is strongly expressed by macrophages and is involved in apoptotic cell clearance,^23^ (2) it is upregulated in SS-TAMs,^6^ (3) it regulates alternative macrophage activation^24^ and may therefore contribute to protumor macrophage activation and (4) we observed during the present studies that the galectin-3 gene, *Lgals3*, was upregulated when M_(IFN-*γ*/LPS)_ macrophages were cocultured with apoptotic cells (Figure 3b). Galectin-3-deficient M_(IFN-*γ*/LPS)_ macrophages proved to be less effective than wild-type (WT) counterparts in antilymphoma activity, but were comparable to WT macrophages in the promotion of lymphoma growth stimulated by apoptotic tumor cells. Similar observations were made both with human and murine lymphoma preparations (Figures 5a and b). These results suggest that galectin-3 is involved in the control of macrophage antitumor activity, but is dispensable for the lymphoma growth-promoting effects of apoptotic cell-triggered M_(IFN-*γ*/LPS)_ macrophages.

### Aggressive lymphoma growth is impaired in mice deficient in galectin-3

**Figure 6:**
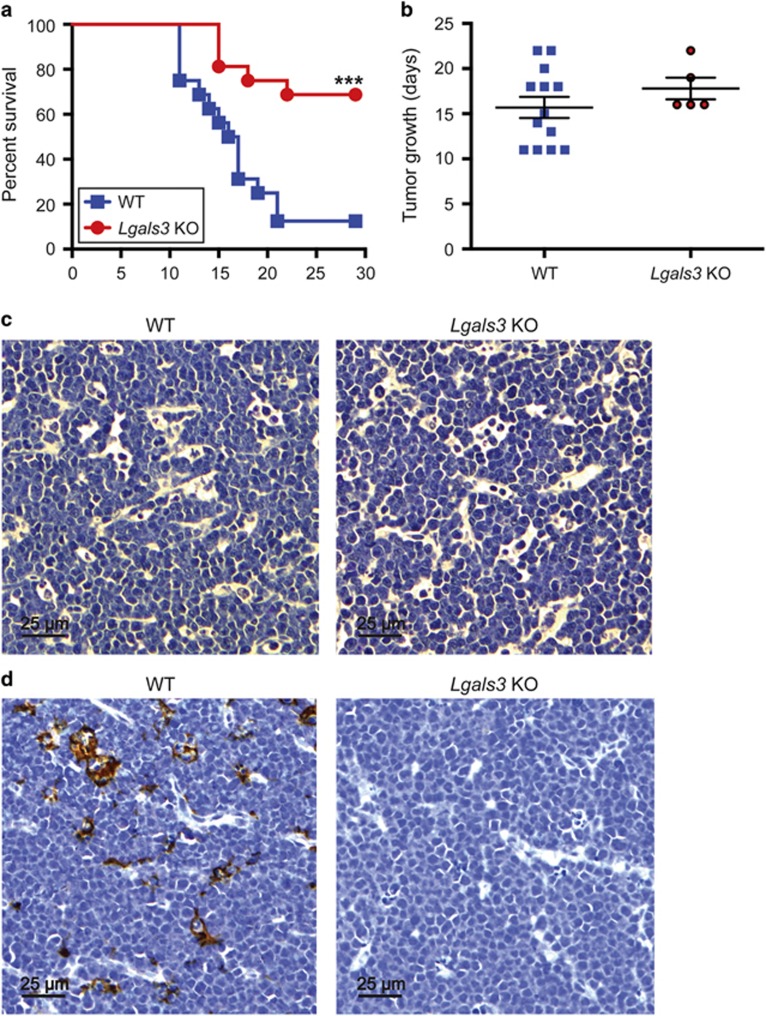
Aggressive lymphoma growth is impaired in mice deficient in galectin-3. Tumor growth in WT C57BL/6 and *Lgals3* C57BL/6 KO mice injected with 0.5 × 10^6^ *λ*-MYC cells. (**a**) Kaplan–Meier plot showing killing following tumor growth to 1.44 cm^2^ (*n*=15 WT and *n*=16 KO mice). ***P<0.001, Mantel-Cox log rank test. (**b**) When a tumor formed, the time taken for the tumors to grow to 1.44 cm^2^±S.E.M. is shown (*n*=13 WT and *n*=5 KO mice). No statistically significant difference. (**c**) Representative hematoxylin and eosin staining of *λ*-MYC tumors grown in WT (*n*=12) or *Lgals3* KO (*n*=5) mice. (**d**) Representative galectin-3 immunohistochemistry of *λ*-MYC tumors grown in WT (*n*=12) or *Lgals3* KO (*n*=5) mice. Strong staining of galectin-3 in WT tumors is associated with TAMs. Note there is also some indication of possible low-level staining by tumor cells

Impairment of the antitumor capacity of galectin-3-deficient M_(IFN-*γ*/LPS)_ macrophages *in vitro* suggested that galectin-3 may be important in innate antitumor immunity in NHL as has been indicated in other models.^25, 26^ Conversely, the upregulation of galectin-3 gene expression in SS-TAMs may support the argument that galectin-3 imparts protumor activity. To clarify the potential pro- or antitumor properties of galectin-3 in NHL, we next determined the effect of galectin-3 deficiency in the λ-MYC model of aggressive B-cell lymphoma.^6^ As shown in Figure 6a, galectin-3-deficient mice developed tumors less frequently compared with their WT counterparts. In cases where tumors developed successfully in galectin-3 knockout (KO) animals, neither growth rate nor histological architecture were significantly altered by the absence of galectin-3 (Figures 6b and c). Note that, in the WT animals, galectin-3 was expressed prominently by TAMs but not by lymphoma cells (Figure 6d). Taken together with the *in vitro* observations, the general suppression of tumor growth in the absence of galectin-3 *in vivo* suggests (1) that host galectin-3-mediated antilymphoma activity is required to sustain net tumor growth and/or (2) that additional function(s) of galectin-3 drive key pro-oncogenic mechanisms in NHL.

## Discussion

Here we have considered the inter-relationships of pro- and antitumor macrophage activities in the context of starry-sky NHL, an aggressive malignant disease in which apoptosis, TAMs and apoptotic cell clearance by TAMs are prominent features. Emerging evidence strongly supports the view that apoptosis of tumor cells can generate protumor signals that polarize TAMs towards a protumor phenotype. We recently showed that protumor TAMs accumulate in starry-sky NHL in response to apoptosis.^6^ Given the possibility that the antitumor potential of TAMs may be harnessed or stimulated for improved anticancer therapy, we sought to clarify the effects of apoptotic lymphoma cells on antitumor TAMs.

We were initially interested in determining the extent to which characteristics of classical macrophage activation – which is known to lead to antitumor activity – could be observed in macrophages interacting with apoptotic cells in the tumor microenvironment. We previously noted that microdissected TAMs accumulating in starry-sky NHL (SS-TAMs), defined by their capacity to engulf apoptotic tumor cells, displayed features of classically activated macrophages alongside their prominent protumor characteristics.^6^ Here we analyzed the classically activated features of SS-TAMs in more detail and compared these with other monocyte and macrophage data sets, including alternative TAMs. We found that the majority of these cell populations exhibited features typical of the classical IFN/LPS response. This finding may not be surprising, given that features of the classical activation state merely provide descriptive indicators of various states of macrophage polarization in an activation continuum rather than definitive activation signatures or lineage paths.^27^ Thus, although the classical activation stimulus (IFN-*γ* and LPS) may be regarded as prototypically antitumor, some of the characteristics of this polarization may feature as part of the activation profile of protumor TAMs. In the specific case of SS-TAMs, these macrophages were found to display multiple features typical of classically activated, antitumor macrophages despite engagement in the clearance of apoptotic cells, which are known to have reparatory stimulatory activities,^4, 28^ and which would be expected as a consequence to provide protumor signals. It is possible that the combination of seemingly pro- and antitumor features displayed by SS-TAMs represents the ‘yin and yang' of the tumor microenvironment: innate antitumor properties of host macrophages being tempered by exposure to (apoptotic) tumor cells (*vide infra*). In established tumors, this interplay may stem from the mechanisms through which the antitumor phenotype of early macrophages is inhibited in nascent tumors and our observations support the contention that apoptosis is an important inhibitory effector of such innate antitumor immunity.

In accordance with previous studies, endocytic and phagocytic pathways were highly prominent in the transcriptome of all macrophages, including the SS-TAMs, whose main phagocytic substrates are apoptotic tumor cells. In the context of normal tissue turnover, clearance of apoptotic cells is a homeostatic mechanism, mediated by tissue-resident macrophages (as well as non-macrophage phagocytic neighbors) and known to drive a reparatory, anti-inflammatory phenotype, for example, in normal germinal centers.^6^ Here we demonstrate that classically activated macrophages display enhanced phagocytosis of apoptotic lymphoma cells (Figure 2b). Our results suggest that in an inflammatory context – such as during infection, wounding or in malignant tissue – classical polarization of macrophage activation would endow responding macrophages with enhanced capacity to engulf apoptotic cells.

In addition to switching on their phagocytic program, macrophages engage in a spectrum of activities in response to apoptotic cells, which collectively foster the resolution of inflammation, the sculpting of organs during development, the promotion of angiogenesis and wound repair. All these macrophage activities are features of TAMs and indicate that apoptosis could provide critical signals to control the activation of macrophages in the tumor microenvironment. Here we have extended this notion to show that apoptotic lymphoma cells dominantly change the activation status of macrophages from anti- to protumor at the transcriptional level. These findings accord with previous studies that demonstrated that preincubation with apoptotic cells can increase anti-inflammatory and decrease proinflammatory signaling by monocytes and macrophages.^29, 30^ Notably, the transcriptional switching we observed was, at least in part, independent of direct contact with apoptotic cells, indicating that these macrophage responses can be uncoupled from phagocytosis. The underlying molecular mechanisms remain to be determined and may involve the release of intercellular signaling moieties from the apoptotic cells in soluble and/or membrane-bound, vesicular forms. These results suggest that the macrophage response to apoptotic lymphoma cells may extend beyond that of the immediate phagocyte. Candidate molecules released from apoptotic cells that are known to signal macrophage responses include lysophosphatidylcholine, sphingosine-1-phosphate, fractalkine and ATP,^28, 31, 32, 33^ but apoptosis yields a broadening spectrum of biologically active factors,^34^ which could theoretically contribute to the transcriptional switching of responding macrophages and elucidation of the active components will require extensive future investigation.

In concert with the changes in activation profile, we found M_(IFN-*γ*/LPS)_ macrophages exposed to apoptotic lymphoma cells to be functionally altered, becoming compromised in their capacity to kill tumor cells. These observations extend those reported in a previous study in which apoptotic cells appeared to reduce cytotoxicity of classically activated macrophages, although it was previously unclear whether the modulatory effects of the apoptotic cells resulted from their interaction with macrophages or with LPS.^22^ Our results also extend the work of Evans and Alexander who, as early as 1970, noted that exposure of lymphoma cells to cytotoxic macrophages inhibited the latter cells' ability to exert antitumor effects.^35^ We were surprised to observe, in addition, that M_(IFN-*γ*/LPS)_ macrophages treated with apoptotic lymphoma cells were endowed with the capacity to promote overall expansion of lymphoma cell populations. Reflecting the dynamics of lymphoma growth in starry-sky tumors, cocultures of apoptotic cell-treated M_(IFN-*γ*/LPS)_ macrophages displayed net total population growth in the face of substantial apoptosis (Figures 4b and c). This effect appeared to be mediated through cell-to-cell contact, although relatively large (>400 nm diameter) extracellular vesicles may also have a role. Thus, macrophages initially exhibiting a classical antitumor activation profile, can gain protumor characteristics upon exposure to apoptotic tumor cells. This reprogramming may reflect the sequence of activation steps undertaken by TAMs, suggesting a basis for the combined pro- and antitumor features displayed by SS-TAMs *in situ*.

**Figure 7:**
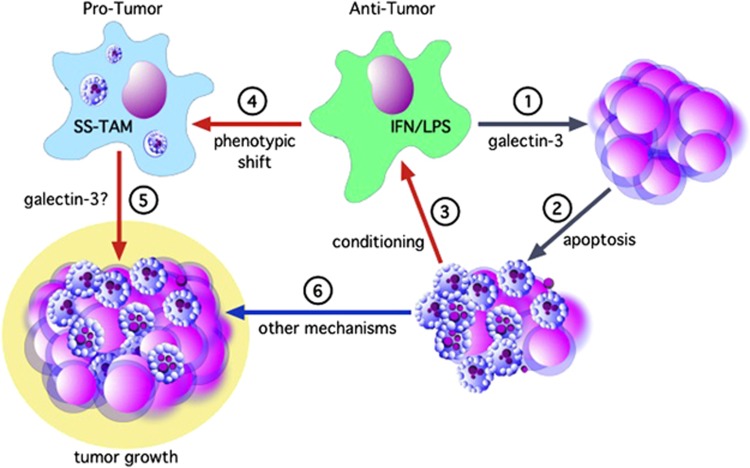
Schematic outline of possible inter-relationships between macrophages and (apoptotic) lymphoma cells that lead to net tumor growth. Through pathway 1, activity of highly phagocytic antitumor macrophages (green), typified in these studies by M_(IFN-*γ*/LPS)_ macrophages, causes apoptosis (2) in lymphoma cells. This displays galectin-3-dependence. Apoptotic cells in the lymphoma cell population—produced by pathway 1,2 or other mechanisms—are able to condition macrophages (3), at least partially without the need for direct contact. This apoptotic cell conditioning causes a phenotypic shift in macrophage activation (4) to a tumor-promoting phenotype typical of SS-TAMs which promote tumor growth (5) via multiple mechanisms as previously described.^6^ This activity may also be galectin-3-dependent. Other mechanisms through which apoptotic lymphoma cells promote net tumor growth independently of macrophages may also be important (6). The antitumor activity of macrophages represented here could be inherent in (certain) TAMs, especially at early stages of tumor development and/or could by induced by macrophage-activating therapies

Although galectin-3 was rationalized as a promising candidate molecular player in the modulation of macrophage activation by apoptotic cells and consequent promotion of lymphoma growth, we found that it had no part in this process, at least in our *in vitro* model. Rather, our results demonstrated a novel activity of galectin-3 in macrophage cytotoxicity. However, NHL growth in mice was substantially inhibited by global galectin-3 deficiency. Given that galectin-3 appears largely restricted to SS-TAMs in these tumors (although it remains possible that galectin-3 expressed at low levels by tumor cells, and other stromal cells may also contribute), these results suggest that galectin-3 is closely linked to protumor functional activation in SS-TAMs. Possible mechanistic scenarios are summarized in Figure 7. Assuming its main functional activities in this context are via TAMs, we propose a dynamic process in which galectin-3 contributes to apoptosis induced in the tumor cell population by antitumor macrophage activity. This apoptosis (alongside the consequences of additional proapoptosis signals to tumor cells, such as nutrient insufficiency^34^) leads to conditioning of the antitumor macrophages by the apoptotic cells and resultant shift in activation profile of SS-TAMs to a protumor phenotype. Additional possible galectin-3 activities *in vivo* remain to be determined, but the results obtained thus far provide strong evidence for targeting galectin-3 in NHL therapy.

In conclusion, although repolarization of TAMs to an antitumor phenotype provides a plausible route to improving antitumor therapies, its successful clinical application requires that the environmental signals to which the macrophage responds be carefully considered.^36^ Our results provide a cautionary note to this approach, indicating that constitutive apoptosis in the tumor or apoptosis induced by repolarized, cytotoxic TAMs or by combination radiation or chemotherapy could militate against therapeutic success by contributing to what has been aptly termed ‘a misdirected tissue repair response'.^2^ Further studies are required to identify the mechanisms responsible for the reprogramming of cytotoxic TAMs by apoptotic tumor cells as these could provide critical therapeutic targets.

## Materials and methods

### *In situ* transcriptomic and bioinformatic analyses

Detailed SS-TAM and GCM *in situ* gene expression profiling is described in our previous work.^6^ In brief, laser capture microdissection was performed on frozen 10 *μ*m SCID/BL2 tumor xenograft tissue sections stained with CD68-AlexaFluor 488 antibody (Life Technologies, Paisley, UK; MCA1957A488) with Protector RNase Inhibitor (4 U/ml; Roche) or tissue sections of splenic germinal centers from CSF1R EGFP transgenic BALb/c mice stimulated with two doses of 5 × 10^6^ sheep red blood cells. Captured cells (1000 cells) were lysed and RNA extracted, amplified and cDNA synthesized and processed for gene microarray hybridization on Affymetrix Mouse Gene 1.0 GeneChip arrays (Affymetrix, High Wycombe, UK). Background subtraction and normalization were performed using the robust multichip average (RMA) algorithm in the OneChannelGui package. Statistical analysis was conducted using the LIMMA software Bioconductor (http://www.bioconductor.org) and the Benjamini and Hochberg procedure was used for multiple testing corrections. Genes with a corrected *P*-value <0.05 and an absolute fold change of 1.8 or greater were considered to be differentially expressed.

Monocyte, macrophage and dendritic cell expression data sets were searched on the National Center for Biotechnology Information's GEO (http://www.ncbi.nlm.nih.gov/geo/). Data sets were selected based on the following criteria: (1) chip platform (Affymetrix Mouse Gene 1.0 ST Array, Affymetrix, High Wycombe, UK), (2) cell type studied; (3) availability of raw data (.cel) files and (4) availability of at least two replicates of each cell type within each study. Compatibility of the raw data was assessed using the arrayQualityMetrics package from Bioconductor. The data were scored on the basis of seven metrics. Any array suggested to be an outlier on more than one metric was removed from the data set. Furthermore, data sets that comprised extra time-points, treatments or knockouts were removed to simplify the data set. Each remaining data set was then normalized independently, using the RMA expression measure,^37^ and any outlier data sets were removed. Probesets of the microarrays were annotated using the latest annotation available from Bioconductor (March 2014) to link the AffyIDs to better understandable gene names. Using a Pearson's threshold cutoff of *r*=0.80, a network graph was generated of the data using BioLayout Express3D (www.biolayout.org).^38^ Using the MCL algorithm, the graph was then clustered non-subjectively into clusters of genes that share similarities in their expression, with the MCL inflation value set to 2.2. For the largest clusters and other clusters of interest, the gene transcripts were analyzed for functions using the Functional Annotation Clustering tool on the Database for Annotation, Visualization, and Integrated Discovery (DAVID; http://david.abcc.ncifcrf.gov/).^39, 40^ Additionally, genes found to be upregulated by SS-TAMs (compared with GCMs and lymph node macrophages (LNMs))^6^ (GEO accession number GSE64366) were overlaid on the network graph to identify the gene clusters with which they were associated.

### Cells and tumors

BMDMs were prepared from the femurs of 8–24-week-old Balb/c, C57BL/6 or C57BL/6 *Lgals3* KO female mice, and cultured for 7–8 days in complete RPMI medium (Gibco RPMI-1640; Life Technologies) supplemented with 10% fetal bovine serum (Biosera Ltd, Ringmer, UK), 2 mM l-glutamine (Life Technologies), 100 U/ml penicillin and 100 *μ*g/ml streptomycin (GE Healthcare Life Sciences, Buckinghamshire, UK) and 100 ng/ml rhM-CSF (R&D Systems, Abington, UK) on bacteriological-grade Petri dishes (Sterilin; Fisher Scientific Ltd, Loughborough, UK). This generated cells that were >97% CD11b and F4/80 positive as measured by flow cytometry. Cells were detached from dishes by spraying with an 18 g needle and 20 ml syringe. In some *in vitro* cocultures, BMDMs were classically activated using 10 U/ml murine IFN-*γ* (R&D Systems) and 0.5 ng/ml LPS (Sigma-Alrich, St. Louis MO, USA; (rough strains) from *S. enterica* serotype Minnesota Re595) for 4 h before coculture. The human (EBV-negative) BL cell lines BL2 and BL2-Bcl-2 (BL2 cells stably transfected with *Bcl-2* to suppress apoptosis)^18^ were cultured in suspension in 50% X-VIVO medium (50% Gibco RPMI-1640, 50% X-VIVO-20 medium; Lonza, Basel, Switzerland), supplemented with 50 U/ml penicillin and 50 *μ*g/ml streptomycin at 37 °C with 5% CO_2_. Bcl-2 protein expression was regularly tested by flow cytometry and >98% of the BL2-Bcl-2 cells expressed Bcl-2. *λ*-MYC cells were derived and cultured as described.^6^ Tumors were generated by subcutaneous injection of 6–12-week-old male C57BL/6 mice (WT or *Lgals3* KO) with 5 × 10^5^ *λ*-MYC cells. Growth of tumors was monitored using calipers. In all experiments, mice were humanely killed when tumors reached dimensions equivalent to 1.44 cm^2^. All animal procedures and husbandry were performed under a license from the UK Home Office according to regulations described in the Animals (Scientific Procedures) Act 1986. For animal studies, sample sizes were guided by previous experimentation.^6^

### Induction of apoptosis by UV irradiation

Apoptosis was induced by exposing BL2 or *λ*-MYC cells in 50% X-VIVO medium to 100 mJ/cm^2^ of UVB light using the UVIcab CV006 minicabinet (UVItec Ltd, Cambridge, UK). Following exposure to UVB, cells were returned to the incubator (37 °C, 5% CO_2_) for 2 (*λ*-MYC cells) or 3 h (BL2 cells) before being used in *in vitro* macrophage-tumor cell cocultures or cytotoxicity assays and for 20 h before being used in interaction and phagocytosis assays. Cell viability was assessed by Annexin V/propidum iodide (AxV/PI) staining, using AxV-AlexaFluor-488 (Life Technologies; A13201) with the Attune Acoustic Focusing Cytometer and software (Life Technologies). Alternatively, cells were fixed in 1% (v/v) formaldehyde (Fisher Scientific Ltd) and stained with 250 ng/ml 4′,6-diamidino-2-phenylindole (DAPI) for 2 h before transferring to a slide for visualization under UV illumination at × 400 magnification using an Axiovert 25 inverted microscope (Zeiss, Cambridge, UK). Images were recorded with a DFC425C camera with LASv3.8 software (Leica, Milton Keynes, UK).

### Microscopy-based interaction and phagocytosis assay

BL2 or *λ*-MYC cells were induced to undergo apoptosis by exposure to UVB irradiation, followed by a 20 h culture at 37 °C with 5% CO_2_. Mature BMDMs (40 000 cells) were added to each well of a glass slide (Hendley, Loughton, Essex, UK) and incubated for 4 h. After a 20h incubation, 400 000 UVB-irradiated BL2 cells were added to each well or media alone was added and the slides were incubated for 1 h at 37 °C with 5% CO_2_. For interaction assays, non-adherent cells were washed in PBS, before fixing the slides in 100% methanol for 20 min. For phagocytosis assays, washing in PBS was followed by proteolytic removal of apoptotic cells bound to the macrophage surface with trypsin-EDTA (Fischer Scientific Ltd) for 5 min, and the remaining non-phagocytosed cells were washed off with PBS before fixation in methanol. Cells were then stained using May–Grünwald–Giemsa. The number of macrophages interacting with 0, 1 or 2 or more apoptotic cells were then counted for five randomly chosen areas per well (blinded and duplicate wells for each treatment) using an Olympus CX40 microscope (Olympus, Southend-on-Sea, UK), and the mean percentage of macrophages interacting with/phagocytosing apoptotic cells was calculated. Statistics were performed on biological repeats, that is, for each n number, BMDMs were derived from a separate animal.

### Coculture of macrophages and tumor cells

BMDMs were seeded in tissue culture plates. BL2, BL2-Bcl-2 or *λ*-MYC lymphoma cells induced to undergo apoptosis were added to the macrophages at a 1 : 10 macrophage:lymphoma cell ratio. In contact-free cocultures, lymphoma cells were placed on top of a 0.44 *μ*m pore-size Millipore Cell culture plate insert (Sigma-Aldrich). Following a 24 h coculture, supernatants were collected and cleared by sequential 300x*g* and 1200 × *g* centrifugation steps for cytokine analysis. Adherent macrophages were washed repeatedly with PBS to completely remove the lymphoma cells, before RNA was extracted using an RNeasy Mini Kit (Qiagen, Manchester, UK).

### Cytotoxicity assay

For cytotoxicity assays, macrophage and lymphoma cell cocultures were performed as detailed above, but BMDMs were stained with PKH67 Green Fluorescent Cell linker dye (Sigma-Aldrich) before seeding in tissue culture plates in duplicate for each treatment. Following coculture with apoptotic or viable lymphoma cells, macrophages were cocultured at a 1:1 ratio with new (target) BL2 lymphoma cells taken from fresh cultures and stained with PKH26 red fluorescent cell membrane dye (Sigma-Aldrich) in 1% X-VIVO medium (1% X-VIVO-20 medium in 99% Gibco RPMI-1640, supplemented with 100 U/ml penicillin and 100 *μ*g/ml streptomycin). After a 20 h coculture with target BL2 cells, all cells were dislodged from the plates by repeated pipetting, stained with F2N12S and SYTOX AADvanced dead cell stain from the Violet Ratiometric Membrane Asymmetry Probe/Dead Cell Apoptosis Kit (Life Technologies) and analyzed on the Attune Acoustic Focusing Cytometer. Compensation was computed using the cytometer's software. Results were analyzed as follows: a gate was drawn on the FSC/SSC scatter plot to select all cells; cells that were positive for PKH67 green fluorescent marker (macrophages) were subtracted from the total cells; from the remaining cells, red fluorescent-positive cells were selected (PKH26-labeled target cells) and the ratio of orange fluorescence *versus* green fluorescence signal of the F2N12S ratiometric membrane asymmetry probe was assessed for the target cells only. Statistics were performed on biological repeats, that is, for each *n* number, BMDMs were derived from a separate animal.

### Real-time qPCR

Extracted RNA was DNase treated using Amplification Grade DNase 1 (Sigma-Aldrich), and cDNA was generated from total RNA using SuperScript III First-Strand Synthesis SuperMix for qRT-PCR (Life Technologies). Real-time qPCR was performed in duplicates in an ABI 7500 FAST qPCR system using SYBR Green I Dye chemistry (Life Technologies) and gene-specific primers. Primers were ordered from Eurofins MWG Operon (Ebersberg, Germany) and are listed in Supplementary Table S1. Relative mRNA expression for genes of interest was assessed in comparison with control samples, following normalization to the reference genes *α*-tubulin (*Tuba1b*), heat-shock protein 90 (*Hsp90*), hypoxanthine guanine phosphoribosyl transferase (*Hprt*) and *β*2 microglobulin (*B2m*) using the ΔΔCT method (described in Applied Biosystems User Bulletin No. 2 (P/N 4303859)^41^ in Excel). Appropriateness of reference genes was tested using NormFinder (version 20).^42^ The relative quantitation data are presented in graphs showing log_2_ expression ratio relative to control samples with error bars corresponding to the S.E.M. Statistical analysis was performed on the raw (ΔCT) data for biological replicates, that is, for each *n* number, BMDMs were derived from a separate animal.

### Histology

Tissue sections were stained with standard hematoxylin and eosin or were used in immunohistochemistry as described in Truman *et al.*^32^ Formalin-fixed, paraffin-embedded tissue was probed with antibodies to galectin-3 (Bio-Techne, Oxon, UK; clone M3/38) following heat treatment in citric acid-based antigen retrieval solution. Immunohistochemical staining was detected using a biotinylated anti-rat secondary antibody (Vector Laboratories, Peterborough, UK) and 3,3′-diaminobenzidine (Vector Laboratories) with hematoxylin counterstain. Images were acquired using a Zeiss Axioskop 2 microscope (Carl Zeiss, Cambridge, UK), with a x20 objective, a Leica DFC425C camera and LASv3.8 software.

### Statistical analysis

Results from experiments are presented as individual data points or means±S.E.M. Data analysis was performed using the GraphPad Prism version 6 (GraphPad, La Jolla, USA). The following statistical methods were used on data which were assumed to be normally distributed: two-tailed paired or unpaired *t*-test, one-way ANOVA followed by Dunnett's post test or two-way ANOVA followed by Tukey's post test. *P*-values of 0.05 or less were considered statistically significant.

## Figures and Tables

**Table 1:** Selected transcripts associated with classical macrophage activation that are upregulated in SS-TAMs

**Gene**	**Protein**	**Fold change**	***P-*****value**
*Ifit3*	Interferon-induced protein with tetratricopeptide repeats 3	5.8	0.0190023
*Ccl8*	Chemokine (C–C motif) ligand 8	5.6	0.00193649
*Fcgr1*	Fc receptor, IgG, high-affinity I	5.5	0.00481987
*Ccl3*	Chemokine (C–C motif) ligand 3	5.2	0.00108299
*Ifit2*	Interferon-induced protein with tetratricopeptide repeats 2	5.1	0.00354699
*Tlr13*	Toll-like receptor 13	4.3	0.0328242
*Tlr4*	Toll-like receptor 4	4.3	0.0010059
*Tlr8*	Toll-like receptor 8	4.0	0.000454208
*Fcer1g*	Fc receptor, IgE, high-affinity I, *γ* polypeptide	3.8	0.000129844
*Ccl12*	Chemokine (C–C motif) ligand 12	3.8	0.024655
*Fcgr3*	Fc receptor, IgG, low-affinity III	2.3	0.0113843
*Ifitm2*	Interferon-induced transmembrane protein 2	2.3	0.0221287
*Irf7*	Interferon regulatory factor 7	2.1	0.0395954
*Tlr2*	Toll-like receptor 2	2.1	0.00388199
*Ccl7*	Chemokine (C–C motif) ligand 7	2.1	0.000652815
*Fcgrt*	Fc receptor, IgG, *α*-chain transporter	2	0.00946608
*Ccl2*	Chemokine (C–C motif) ligand 2	2	0.0113823

Abbreviations: DAVID, Database for Annotation and Integrated Discovery; GCM, germinal center macrophage; SS-TAM, starry-sky TAM; TAM, tumor-associated macrophage

Functional annotation using DAVID revealed that multiple transcripts associated with classically activated macrophages are upregulated by SS-TAMs, as compared with GCMs. Upregulated genes and fold change compared with GCMs are shown here. Data are from three animals. SS-TAMs were from BL2 xenografts and all transcriptional profiles were derived from laser capture-microdissected macrophages (see Materials and Methods)

## Abbreviations

AxV: Annexin V
BL: Burkitt's lymphoma
BMDMs: bone marrow-derived macrophages
DAPI: 4′,6-diamidino-2-phenylindole
DAVID: Database for Annotation and Integrated Discovery
*Lgals3* KO: galectin-3 knockout
GCM: germinal center macrophage
GEO: Gene Expression Omnibus
IFN: interferon
LNM: lymph node macrophage
LPS: lipopolysaccharide
M_(IFN-*γ*/LPS)_: IFN-*γ* and LPS (classically) activated macrophages
MCL: Markov Clustering
NHL: non-Hodgkin's lymphoma
PI: propidium iodide
rhM-CSF: recombinant human macrophage colony-stimulating factor
RMA: robust multiarray average
SS-TAM: starry-sky tumor-associated macrophage
TAM: tumor-associated macrophage
