# Supplementary material for: Modulation of macrophage antitumor potential by apoptotic lymphoma cells
Source: Cell Death Differ. 2017 Feb 3;24(6):971–83. doi: 10.1038/cdd.2016.132 (PMC5442466; doi:10.1038/cdd.2016.132)
Supplement: Supplementary Table S1 [file cdd2016132x1.docx]

| Gene | Genbank accession | Primer sequence (5’ ➞ 3’) | Amplicon size (bp) | Slope | R^2^ |
| --- | --- | --- | --- | --- | --- |
| Abca1 | NM_013454 | F: AAAACCGCAGACATCCTTCAG  R: CATACCGAAACTCGTTCACCC | 124 | -3.26 | 0.97 |
| Anpep | NM_008486 | F: ATGGAAGGAGGCGTCAAGAAA  R: CGGATAGGGCTTGGACTCTTT | 180 | -3.34 | 0.98 |
| Arg1 | NM_007482 | F: CTCCAAGCCAAAGTCCTTAGAG  R: AGGAGCTGTCATTAGGGACATC | 185 | -3.26 | 0.98 |
| Axl | NM_009465 | F: ATGGCCGACATTGCCAGTG  R: CGGTAGTAATCCCCGTTGTAGA | 155 | -3.36 | 0.98 |
| B2m | NM_009735 | F: ACCCGCCTCACATTGAAATCC  R: GGCGTATGTATCAGTCTCAGTG | 146 | -3.43 | 0.99 |
| Ccl2 | NM_011333 | F: TTAAAAACCTGGATCGGAACCAA  R: GCATTAGCTTCAGATTTACGGGT | 121 | -3.27 | 0.99 |
| Cd36 | NM_007643 | F: AGATGACGTGGCAAAGAACAG  R: CCTTGGCTAGATAACGAACTCTG | 83 | -3.35 | 0.99 |
| Cd68 | NM_009853 | F: CCTCGCCTAGTCCAAGGTC  R: GGATTCGGATTTGAATTTGGGCT | 102 | -3.44 | 0.99 |
| Cd93 | NM_010740 | F: ATCTCAACTGGTTTGTTCCTGC  R: ACTCTTCACGGTGGCAAGATT | 186 | -3.55 | 0.99 |
| Csf1r | NM_001037859 | F: GGACCTACCGTTGTACCGAG  R: CAAGAGTGGGCCGGATCTTT | 85 | -3.34 | 0.99 |
| Ctsb | NM_007798 | F: TCCTTGATCCTTCTTTCTTGCC  R: ACAGTGCCACACAGCTTCTTC | 176 | -3.50 | 0.99 |
| Ctsd | NM_009983 | F: GCTTCCGGTCTTTGACAACCT  R: CACCAAGCATTAGTTCTCCTCC | 113 | -3.53 | 0.99 |
| Ctsl | NM_009984 | F: ATCAAACCTTTAGTGCAGAGTGG  R: CTGTATTCCCCGTTGTGTAGC | 136 | -3.38 | 0.99 |
| Ctss | NM_021281 | F: CCATTGGGATCTCTGGAAGAAAA  R: TCATGCCCACTTGGTAGGTAT | 155 | -3.35 | 0.97 |
| Emp1 | NM_010128 | F: TTGGTGCTACTGGCTGGTCT  R: CATTGCCGTAGGACAGGGAG | 169 | -3.58 | 0.99 |
| Emr1 | NM_010130 | F: TGACTCACCTTGTGGTCCTAA  R: CTTCCCAGAATCCAGTCTTTCC | 111 | -3.38 | 0.99 |
| Fn1 | NM_010233 | F: TTCAAGTGTGATCCCCATGAAG  R: CAGGTCTACGGCAGTTGTCA | 154 | -3.49 | 0.99 |
| Gas6 | NM_019521 | F: TGCTGGCTTCCGAGTCTTC  R: CGGGGTCGTTCTCGAACAC | 186 | -3.40 | 0.96 |
| Gpnmb | NM_053110 | F: TGCCAAGCGATTTCGTGATGT  R: GCCACGTAATTGGTTGTGCTC | 76 | -3.11 | 0.99 |
| Hmox1 | NM_010442 | F: AAGCCGAGAATGCTGAGTTCA  R: GCCGTGTAGATATGGTACAAGGA | 100 | -3.148 | 0.98 |
| Hprt | NM_013556 | F: TCAGTCAACGGGGACATAAA  R: GGGGCTGTACTGCTTAACCAG | 141 | -3.56 | 0.99 |
| Hsp90ab1 | NM_008302 | F: GTCCGCCGTGTGTTCATCAT  R: GCACTTCTTGACGATGTTCTTGC | 168 | -3.55 | 0.99 |
| Igf1 | NM_010512 | F: GCAACACTCATCCACAATGC  R: AGCTGGACCAGAGACCCTTT | 148 | -3.53 | 0.99 |
| Il12a | NM_008351 | F: CAATCACGCTACCTCCTCTTTT  R: CAGCAGTGCAGGAATAATGTTTC | 181 | -3.11 | 0.99 |
| Il6 | NM_031168 | F: TAGTCCTTCCTACCCCAATTTCC  R: TTGGTCCTTAGCCACTCCTTC | 76 | -3.25 | 0.99 |
| Lamp2 | NM_010685 | F: TGGCTCAGCTTTCAACATTTCC  R: TGCCAATTAGGTAAGCAATCACT | 278 | -3.56 | 0.98 |
| Lgals3 | NM_010705 | F: GTACAGCTAGCGGAGCGG  R: CGGATATCCTTGAGGGTTTG | 110 | -3.18 | 0.99 |
| Lrp1 | NM_008512 | F: ACTATGGATGCCCCTAAAACTTG  R: GCAATCTCTTTCACCGTCACA | 102 | -3.28 | 0.98 |
| Mertk | NM_008587 | F: CTCCTGAGCCCGTCAATATCT  R: AGACCAGGTACGGTTAGGACA | 94 | -3.10 | 0.99 |
| Mmp12 | NM_008605 | F: TTTGGATTATTGGAATGCTGC  R: ATGAGGCAGAAACGTGGACT | 106 | -3.488 | 0.98 |
| Mmp2 | NM_008610 | F: CAAGTTCCCCGGCGATGTC  R: TTCTGGTCAAGGTCACCTGTC | 171 | -3.51 | 0.99 |
| Mmp3 | NM_010809 | F: TCTGGGCTATACGAGGGCAC  R: ACCCTTGAGTCAACACCTGGA | 232 | -3.19 | 0.96 |
| Mrc1 | NM_008625 | F: CTCTGTTCAGCTATTGGACGC  R: CGGAATTTCTGGGATTCAGCTTC | 132 | -3.599 | 0.98 |
| Msr1 | NM_031195 | F: TTCAAACTCAAAAGCCGACCT  R: GTTGCCATGCTGAAATTCTGG | 60 | -3.29 | 0.98 |
| Nos2 | NM_010927 | F: ACATCGACCCGTCCACAGTAT  R: CAGAGGGGTAGGCTTGTCTC | 177 | -3.85 | 0.99 |
| Pdgfc | NM_019971 | F: ACATTTGATGAGAGATTTGGGCT  R: CAGCGTCCTAAAACACTTCCAT | 104 | -3.2 | 0.99 |
| Plau | NM_008873 | F: GCGCCTTGGTGGTGAAAAAC  R: TTGTAGGACACGCATACACCT | 100 | -3.35 | 0.99 |
| Pparg | NM_011446 | F: GGAAGACCACTCGCATTCCTT  R: GTAATCAGCAACCATTGGGTCA | 121 | -3.33 | 0.99 |
| Psap | NM_011179 | F: CCTGTCCAAGACCCGAAGAC  R: CAAGGAAGGGATTTCGCTGTG | 137 | -3.40 | 0.99 |
| Timp2 | NM_011594 | F: TCAGAGCCAAAGCAGTGAGC  R: GCCGTGTAGATAAACTCGATGTC | 142 | -3.34 | 0.99 |
| Tnf | NM_013693 | F: CAGGCGGTGCCTATGTCTC  R: CGATCACCCCGAAGTTCAGTAG | 89 | -3.19 | 0.99 |
| Trem2 | NM_031254 | F: CTGGAACCGTCACCATCACTC  R: CGAAACTCGATGACTCCTCGG | 183 | -3.45 | 0.99 |
| Tuba1b | NM_011654 | F: AGTAGAGCTCCCAGCAGGC  R: TCTCACCCTCGCCTTCTAAC | 104 | -3.38 | 0.99 |

- - - - 2. Table S1 Sequences and amplification efficiency of mouse primers used in real-time qPCR

Gene symbols, Genbank IDs, forward (F) and reverse (R) oligonucleotide sequences and amplicon size in base pairs (bp) are shown. Amplification efficiency is presented in terms of standard curve slope and R^2^ values.
